# Prevalence and Prognostic Significance of Hyponatremia in Patients with Acute Exacerbation of Chronic Obstructive Pulmonary Disease: Data from the Akershus Cardiac Examination (ACE) 2 Study

**DOI:** 10.1371/journal.pone.0161232

**Published:** 2016-08-16

**Authors:** Jacob A. Winther, Jon Brynildsen, Arne Didrik Høiseth, Ivar Følling, Pål H. Brekke, Geir Christensen, Tor-Arne Hagve, Joseph G. Verbalis, Torbjørn Omland, Helge Røsjø

**Affiliations:** 1 Division of Medicine, Akershus University Hospital, Lørenskog, Norway; 2 Institute of Clinical Medicine, University of Oslo, Oslo, Norway; 3 Institute for Experimental Medical Research, Oslo University Hospital, Ullevål, Oslo, Norway; 4 Division of Diagnostics and Technology, Akershus University Hospital, Lørenskog, Norway; 5 Division of Endocrinology and Metabolism, Georgetown University Medical Center, Washington, DC, United States of America; University of Alabama at Birmingham, UNITED STATES

## Abstract

**Background:**

Hyponatremia is prevalent and associated with mortality in patients with heart failure (HF). The prevalence and prognostic implications of hyponatremia in acute exacerbation of chronic obstructive pulmonary (AECOPD) have not been established.

**Method:**

We included 313 unselected patients with acute dyspnea who were categorized by etiology of dyspnea according to established guidelines (derivation cohort). Serum Na^+^ was determined on hospital admission and corrected for hyperglycemia, and hyponatremia was defined as [Na^+^]<137 mmol/L. Survival was ascertained after a median follow-up of 816 days and outcome was analyzed in acute HF (n = 143) and AECOPD (n = 83) separately. Results were confirmed in an independent AECOPD validation cohort (n = 99).

**Results:**

In the derivation cohort, median serum Na^+^ was lower in AECOPD vs. acute HF (138.5 [135.9–140.5] vs. 139.2 [136.7–141.3] mmol/L, p = 0.02), while prevalence of hyponatremia (27% [22/83] vs. 20% [29/143], p = 0.28) and mortality rate (42% [35/83] vs. 46% [66/143], p = 0.56) were similar. By univariate Cox regression analysis, hyponatremia was associated with increased mortality in acute HF (HR 1.85 [95% CI 1.08, 3.16], p = 0.02), but not in AECOPD (HR 1.00 [0.47, 2.15], p = 1.00). Analogous to the results of the derivation cohort, hyponatremia was prevalent also in the AECOPD validation cohort (25% [25/99]), but not associated with mortality. The diverging effect of hyponatremia on outcome between AECOPD and acute HF was statistically significant (p = 0.04).

**Conclusion:**

Hyponatremia is prevalent in patients with acute HF and AECOPD, but is associated with mortality in patients with acute HF only.

## Introduction

Hyponatremia is observed in a wide variety of medical disorders, and it is the most frequent electrolyte disturbance encountered in clinical practice [1]. In one large study of hospitalized patients, hyponatremia, defined as [Na^+^] below 136 mmol/L, was found in 28% of subjects, and the risk of hyponatremia increased with age [2]. In heart failure (HF), hyponatremia has been reported to be prevalent in both decompensated (20–27%) [3, 4] and stable disease (17%) [5]. In contrast, limited information is available in the literature regarding the prevalence of hyponatremia in patients with chronic obstructive pulmonary disease (COPD).

Hyponatremia correlates with prognosis in various populations. In a cross-sectional study of adults, subjects with hyponatremia had increased risk of mortality compared to subjects with normal Na^+^ levels after adjustment for other risk indices [6]. A large study of unselected hospitalized patients also found low admission plasma Na^+^ to be a strong predictor of mortality, including Na^+^ levels within lower reference range (≤138 mmol/L) [7].

Association between hyponatremia and clinical outcome has also been reported in patients with specific disease entities. HF patients with hyponatremia have increased short- [8] and long-term mortality [9] compared to normonatremic HF patients regardless of left ventricular ejection fraction (LVEF) [3, 10, 11]. Even Na^+^ levels in the lower reference range as compared to the upper reference range appear to be associated with worse prognosis in HF [10]. The increased mortality associated with hyponatremia seems at least partly reversible, as patients who improved their Na^+^ levels during follow-up had better outcome than patients with consistently low or declining Na^+^ levels in HF [12] and across a spectrum of conditions as reported in a recent meta-analysis [13]. Strong associations between hyponatremia and mortality have also been demonstrated in liver cirrhosis [14–16], pneumonia [17], and acquired immunodeficiency syndrome [18], but no information is currently available regarding the prognostic significance of hyponatremia in patients with COPD.

Accordingly, as identifying relevant prognostic factors is critical for guiding therapy and may improve our understanding of the pathogenic mechanism of COPD, in this study we hypothesized that hyponatremia is prevalent and associated with outcome in patients with acute exacerbation of COPD (AECOPD). To this end, we explored hyponatremia in two independent AECOPD cohorts and contrasted the results to data obtained in patients with acute HF.

## Subjects and Methods

Detailed information is available in the online supplemental text file (S1 Text).

### Validation and derivation cohort

We included two independent cohorts to explore the association between hyponatremia and outcome in AECOPD. First, we explored the prevalence and association between hyponatremia and outcome in patients with AECOPD and other causes of acute dyspnea in the Akershus Cardiac Examination (ACE) 2 Study (derivation cohort), and then we confirmed the results in an independent AECOPD validation cohort. Finally, to increase the probability of detecting small-scale effects of hyponatremia in AECOPD, we increased the sample size by merging comparable data from the derivation and validation cohort.

**Fig 1:**
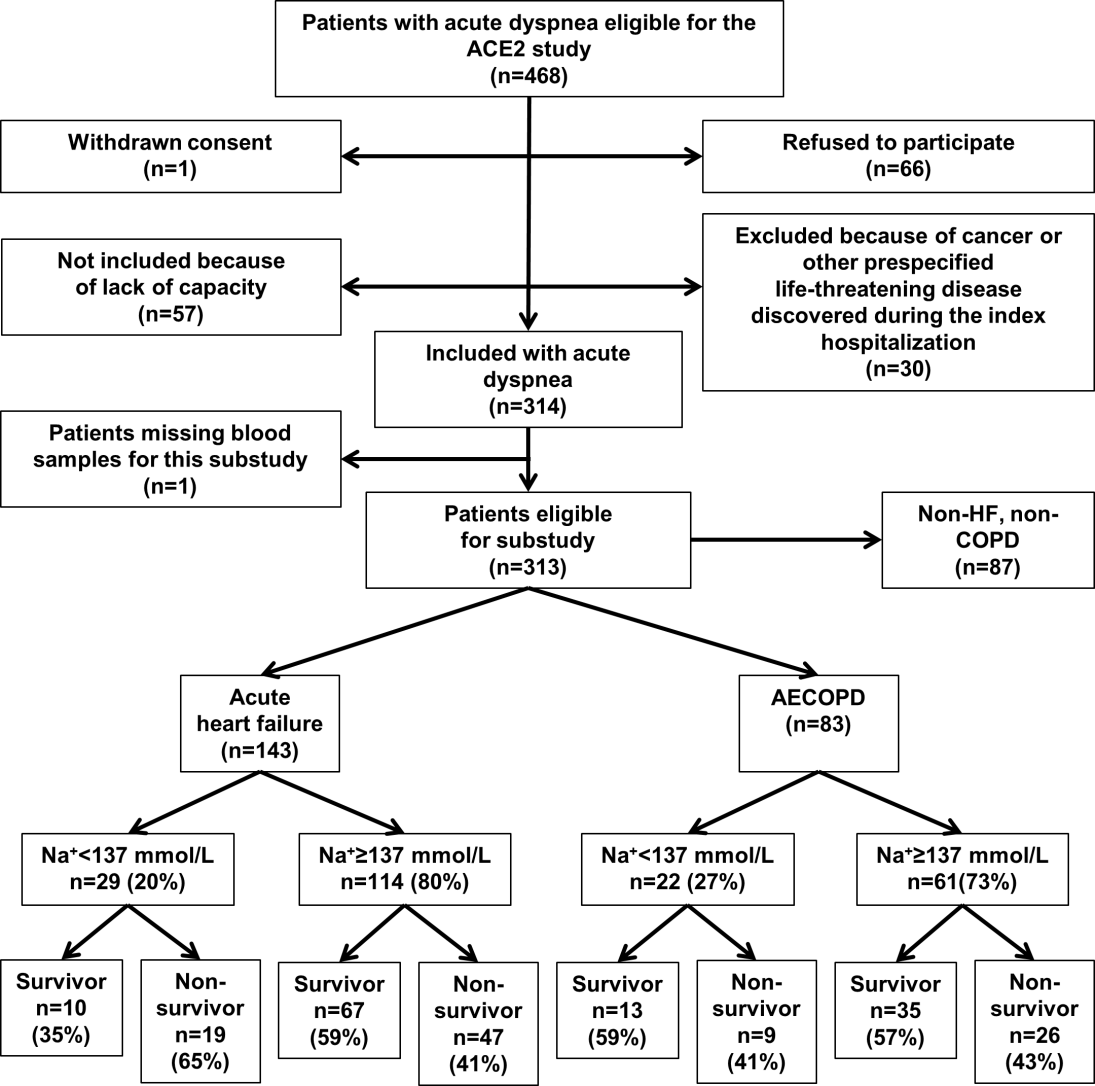
Derivation cohort (ACE 2 study) flow chart.

The ACE 2 Study was designed to study biomarkers in patients admitted with acute dyspnea at Akershus University Hospital, Lørenskog, Norway [19]. In total, 314 subjects were enrolled between June 2009 and November 2010, and for this substudy we included the 313 patients who had Na^+^ measured on admission (Fig 1). Clinical information was obtained from physicians on call, hospital records, and directly from the patients by dedicated study personnel who used standardized questionnaires. Echocardiography and spirometry results were recorded from hospital records. The subjects were categorized into 3 groups according to the final diagnoses of the index hospitalization (Fig 1): Group A. Acute HF (n = 143), group B. AECOPD (n = 83), and group C. Non-HF, non-COPD (n = 87). The diagnoses were adjudicated by two independent senior physicians, who reviewed all medical records, including follow-up data with a median time from hospitalization to adjudication of diagnosis of 464 days (quartile [Q] 1–3 304–705). The acute HF diagnosis was based on the European Society of Cardiology criteria [20] and the diagnosis of AECOPD was based on the criteria defined by the Global initiative for Chronic Obstructive Lung Disease (GOLD) [21]. The two members of the adjudication committee reached the same diagnosis in 95% (298/314) of the cases. Discordant diagnoses were resolved by consensus. Survival status was recorded from electronic hospital records, which are synchronized with Statistics Norway, until the end of follow-up November 1^st^, 2012.

The inclusion strategy of the AECOPD validation cohort has also previously been reported.[22, 23] In short, this study included 99 patients with AECOPD as the admission diagnosis during 2005–2006 at Akershus University Hospital (S1 Fig). Survival status in this cohort was retrieved from the National Population Registry with follow-up ending December 31, 2008. The ACE 2 Study and the validation cohort study were conducted 4 years apart, but the duration of observation was similar. All subjects were admitted to the same center and Na^+^ was routinely measured at admission and analyzed by the same laboratory in both cohorts. Thus, admission Na^+^ measures and survival data were considered comparable between the two cohorts and included in the merged dataset. The two studies were approved by the Norwegian Regional Committees for Medical and Health Research Ethics (REC) South East (ACE 2 Study: #5.2008.2832), conducted according to the Declaration of Helsinki, and all participants provided written informed consent prior to study commencement.

### Laboratory analysis

Serum Na^+^, K^+^, glucose, creatinine, and C-reactive protein (CRP) were determined on hospital admission by standard biochemical methods. Na^+^ at hospital discharge was also included in a subgroup of patients from the derivation cohort with serial Na^+^ measurements. Creatinine clearance was estimated by the Cockcroft-Gault formula [24]. Arterial blood gas measurements from admission were retrieved from hospital records. Na^+^ concentrations were corrected for the diluting effect of hyperglycemia by the Hillier formula [25], and hyponatremia was defined as [Na^+^] <137 mmol/L according to the local reference based on the Nordic reference interval project (NORIP).[26] N-terminal pro-B-type natriuretic peptide (NT-proBNP) and high-sensitivity cardiac troponin T (hs-TnT) were measured in samples obtained <24 h after hospital admission by commercially available assays (proBNP II assay and Troponin T hs STAT, Roche Diagnostics, Penzberg, Germany).

### Statistical analysis

Continuous variables are reported as mean (± standard deviation [SD]) or median (quartile [Q] 1–3), and differences between groups were compared by Student´s t test or Mann-Whitney U tests as appropriate. Binary data were compared by the Chi-square test and are presented as absolute numbers and percentages. Survival according to Na^+^ level is illustrated by Kaplan-Meier plots and compared by the log-rank test, and also assessed by Cox proportional hazard regression analysis. Hazard ratios (HR) are presented with 95% confidence interval (CI). We assessed the association between hyponatremia and outcome in AECOPD and acute HF separately. We also explored whether the effect of hyponatremia on outcome was significantly different between AECOPD and acute HF in the two cohorts in combination. *P* values <0.05 (two-sided) were considered statistically significant. We performed statistical analyses using SPSS for Windows version 22.0 (SPSS, Chicago, IL) and STATA version 14 (Stata Corp LP, TX, USA).

## Results

### Baseline characteristics

Baseline differences between AECOPD and HF in the derivation cohort were consistent with the respective diagnosis. While all AECOPD subjects had a history of COPD at admission, only 61% of acute HF subjects were previously diagnosed with HF. Age, male to female ratio, and body mass index (BMI) were higher in acute HF relative to AECOPD (Table 1). Mortality, follow-up time, and disease severity, as reflected by NYHA functional class, were comparable between AECOPD and acute HF patients.

**Table 1:** Baseline characteristics for patients admitted to hospital with dyspnea.

	Derivation cohort (ACE 2 study)			Validation cohort
	Acute exacerbation of COPD (n = 83)	Acute heart failure (n = 143)	*P*	Acute exacerbation of COPD (n = 99)
***Clinical findings at admission*:**				
Age (years)	69±9	75±11	<0.001	79±9
Male sex	35 (42%)	90 (63%)	0.002	52 (53%)
Body mass index (Kg/m^2^)	24±6	27±6	0.004	23±5
Heart rate (beats/minute)	97±19	92±26	0.10	101±22
Mean arterial pressure (mmHG)	102±18	104±21	0.50	101±19
Peripheral edema	31 (37%)	77 (54%)	0.02	18 (18%)
Pre-hospital SpO_2_ (%)	87 (79–92) ^†^	87 (83–91) ^‡^	0.98	n.a.
NYHA class IV vs. II-III	47 (57%)	65 (46%)	0.11	n.a.
***Heart and lung function*:**				
LVEF (%)	60 (50–60) ^†^	40 (30–55)	<0.001	n.a.
FEV_1_ (mL)	928±456	n.a.		910±450
FEV_1_% of predicted	38±17	n.a.		37±16
FEV_1_/FVC (%)	47±15 ^†^	n.a.		45±14
***Smoking*:**				
Current	28 (34%)	30 (21%)	0.03	35 (35%)
Previous	52 (63%)	74 (52%)	0.11	62 (63%)
Never	3 (3%)	39 (27%)	<0.001	2 (2%)
***History of*:**				
Diabetes	9 (11%)	43 (30%)	0.001	8 (8%)
Heart failure	9 (11%)	87 (61%)	<0.001	14 (14%)
Coronary artery disease	24 (29%)	78 (55%)	<0.001	27 (27%)
Hypertension	26 (31%)	69 (48%)	0.01	31 (31%)
COPD	83 (100%)	61(43%)	<0.001	99 (100%)
***Medication at admission*:**				
Beta-blocker	31 (37%)	89 (62%)	<0.001	28 (28%)
ACEi/ARB	27 (33%)	87 (61%)	<0.001	23 (23%)
Thiazide diuretic	10 (12%)	18 (13%)	0.91	5 (5%)
Loop diuretic	28 (34%)	97 (68%)	<0.001	21 (21%)
Aldosterone antagonist	6 (7%)	21 (15%)	0.10	4 (4%)
***Laboratory findings at admission*:**				
Arterial pH	7.42 (7.39–7.44)	7.43 (7.40–7.45)^†^	0.06	7.40 (0.08)
Arterial pO_2_ (kPa)	8.1 (6.9–9.0)	9.0 (7.7–10.7)^†^	0.001	8.3 (1.8)
Arterial pCO_2_ (kPa)	5.8 (4.9–6.6)	5.1 (4.4–6.2)^†^	<0.001	6.0 (1.5)
Glucose (mmol/L)	6.7 (5.4–8.2)	6.2 (5.4–8.4)	0.84	6.7±2.1
K^+^ (mmol/L)	4.3±0.5	4.4±0.6	0.67	4.1±0.4
Creatinine clearance (mL/min)	73.8 (62.7–92.3)	58.9 (40.7–82.1)	<0.001	75.7±31.4
C-reactive protein (mg/L)	26 (6–50)	13 (5–35)	0.02	29 (9–73)
NT-proBNP (pg/mL)	391 (171–1013)	3600 (1601–8396)	<0.001	423 (154–1311)
hs-TnT (ng/L)	18.2 (9.8–28.4)	37.9 (21.8–75.3)	<0.001	27 (13–51)
***Na***^***+***^ ***during hospital stay*:**				
Admission Na^+^ (mmol/L) *	138.5 (135.9–140.5)	139.2 (136.7–141.3)	0.02	138.3±4.5
Admission Na^+^ < 137 mmol/L *	22 (27%)	29 (20%)	0.28	25 (25%)
Admission Na^+^ < 130 mmol/L *	5 (6%)	5 (4%)	0.37	7 (7%)
Days to discharge Na^+^	2.5 (1–6)^†^	5 (3–8)^†^	0.002	n.a.
Discharge Na^+^(mmol/L)	137(135–139)^†^	139 (136–141)^†^	0.01	n.a.
Discharge Na^+^ < 137 mmol/L	18 (39%)^†^	26 (26%)^†^	0.09	n.a.
ΔNa^+^ (discharge—admission)	1.9±3.8^†^	0.5±4.1^†^	0.06	n.a.
***Follow-up and mortality*:**				
Length of hospital stay	4 (2–7)	6 (3–9)	0.04	n.a.
Follow-up (days)	866 (407–1031)	776 (246–983)	0.07	817 (227–1127)
All-cause mortality	35 (42%)	66 (46%)	0.56	57 (58%)

Continuous variables are presented as mean ± standard deviation if normally distributed or median (quartile 1–3) if non-normally distributed. Binary variables are presented as absolute numbers and percentages.

^†^ Missing = 10–50%

^‡^ Missing = 51–85%

* Na^+^ corrected for hyperglycemia by the Hillier formula [25].

Abbreviations: ACE 2 Study, Akershus Cardiac Examination 2 Study; ACEi, angiotensin-converting-enzyme inhibitor; ARB, angiotensin II receptor blocker; COPD, chronic obstructive pulmonary disease; FEV1, forced expiratory volume in one second; FVC, forced vital capacity; hs-TnT, high sensitivity troponin T; LVEF, left ventricular ejection fraction; n.a., not applicable (missing data > 85% for FEV1 and FVC in acute HF, data not available from validation cohort); NT-proBNP, N-terminal pro-B-type natriuretic peptide; NYHA, New York Heart Association; pO_2_, partial pressure of oxygen; pCO_2_, partial pressure of carbon dioxide; SpO_2_, peripheral capillary oxygen saturation.

### Prevalence of hyponatremia

Unadjusted serum Na^+^ <137 mmol/L was observed in 70 of 313 subjects in the derivation cohort (22%). Stratified according to diagnosis, the crude rate of hyponatremia was 35% (29/83) in AECOPD vs. 27% (38/143) in acute HF (p = 0.18). After correction for hyperglycemia, Na^+^ was lower in AECOPD compared to acute HF (138.5 [135.9–140.5] vs. 139.2 [136.7–141.3] mmol/L, p = 0.02), but the prevalence of hyponatremia was similar: 27% (22/83) vs. 20% (29/143), respectively, p = 0.28 (Table 1). The prevalence of hyponatremia in the AECOPD validation cohort (25% [25/99]) was also comparable to that of AECOPD subjects in the derivation cohort (p = 0.85 for difference between the cohorts).

### Variables associated with hyponatremia

In the derivation cohort, only history of hypertension was associated with hyponatremia in AECOPD: OR 3.07 (95% CI [1.11 8.49], p<0.03 in multivariate analysis) (S1 Table). This association was also significant after post-hoc adjustment for thiazide therapy alone or diuretic therapy in general. Among HF subjects, only BMI remained significantly associated with hyponatremia in the multivariate model (S1 Table).

### Hyponatremia and prognosis

**Fig 2:**
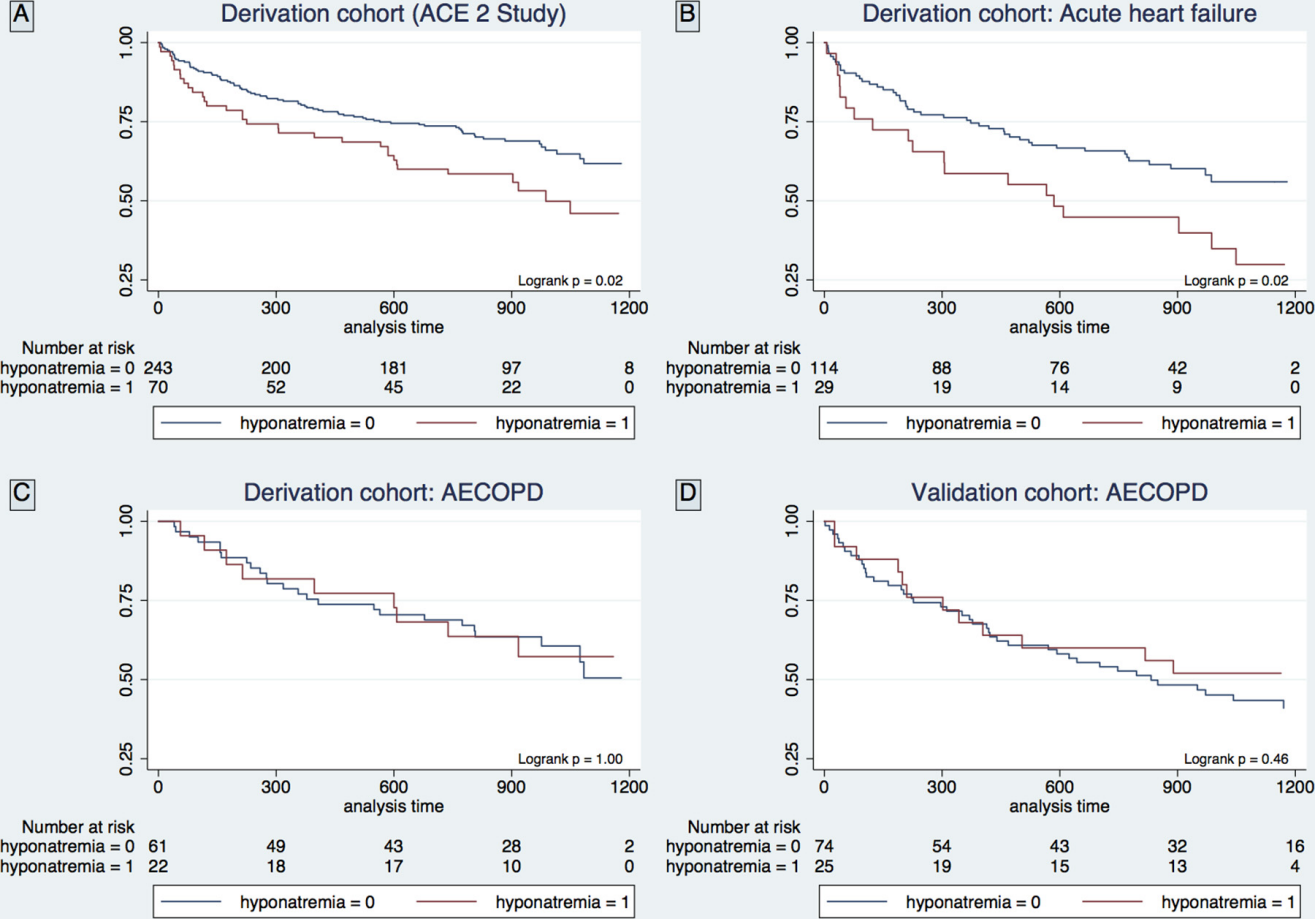
Kaplan-Meier survival plots stratified according to the presence of hyponatremia

Hyponatremia was associated with increased mortality among unselected subjects with acute dyspnea in the derivation cohort (Fig 2A). When subjects were categorized according to the cause of dyspnea, hyponatremia was associated with increased mortality in acute HF (Fig 2B), but not in AECOPD (Fig 2C). The same pattern was evident by univariate Cox regression where hyponatremia was associated with increased the risk of mortality in acute HF (HR 1.85 [95% CI 1.08, 3.16], p = 0.02), but not in AECOPD (HR 1.00 [0.47, 2.15], p = 1.00). Neither discharge Na^+^ nor the change of Na^+^ during admission was associated with morality in acute HF or AECOPD, however, it should be acknowledged that these variables were missing in a proportion of patients. In the AECOPD validation cohort hyponatremia was not associated with mortality as assessed by survival curves (Fig 2D) or Cox regression analysis (HR 0.79 [0.42, 1.49], p = 0.46). Also for the two cohorts in combination, hyponatremia was not associated with mortality by univariate Cox regression analysis in AECOPD (HR 0.87 [0.53, 1.40], p = 0.53), and additionally, the diverging effect of hyponatremia on outcome between AECOPD and acute HF was statistically significant (p = 0.04). Of note, the association between hyponatremia and outcome in acute HF was attenuated and not significant in a comprehensive multivariate model that included established cardiovascular biomarkers (Table 2).

**Table 2:** Cox proportional regression analysis for all-cause mortality in the derivation cohort (ACE 2 study).

	Patients with acute exacerbation of COPD (n = 83)		Patients with acute heart failure (n = 143)	
	Hazard ratio (95% CI)	*P*	Hazard ratio (95% CI)	*P*
***Clinical findings at admission*:**				
Age (years)	1.03 (0.99–1.07)	0.12	1.04 (1.02–1.07)	0.002
Male sex	1.87 (0.96–3.64)	0.07	0.53 (0.32–0.86)	0.01
Body mass index (Kg/m2)	0.90 (0.85–0.97)	0.003	0.94 (0.89–0.99)	0.01
Heart rate (per 5 beats/minute)	1.05 (0.96–1.14)	0.33	0.98 (0.94–1.03)	0.40
Mean arterial pressure (per 5 mmHg)	0.94 (0.84–1.04)	0.23	0.89 (0.83–0.95)	0.001
Peripheral edema	1.11 (0.57–2.19)	0.76	1.33 (0.81–2.17)	0.26
NYHA class IV vs. II-III	1.06 (0.54–2.10)	0.87	2.01 (1.23–3.29)	0.01
***Heart and lung function*:**				
LVEF (%)	0.97 (0.93–1.02)^†^	0.26	1.00 (0.98–1.02)	0.87
FEV_1_ (per 100 mL)	0.95 (0.87–1.03)	0.19	n.a.	
FEV_1_% of predicted (per 10%)	0.79 (0.63–1.00)	0.05	n.a.	
FEV_1_/FVC (per 10%)	0.75 (0.57–0.97)	0.03	n.a.	
***Smoking*:**				
Current vs. previous and never	1.70 (0.85–3.38)	0.13	0.88 (0.48–1.62)	0.68
Never vs. current and previous	n.a.		0.90 (0.52–1.56)	0.70
***History of*:**				
Diabetes mellitus	0.45 (0.11–1.89)	0.28	1.78 (1.08–2.95)	0.02
Heart failure	2.17 (0.83–5.65)	0.11	1.43 (0.86–2.38)	0.17
Coronary artery disease	1.00 (0.48–2.09)	0.99	1.05 (0.65–1.70)	0.85
Hypertension	1.05 (0.51–2.14)	0.90	0.83 (0.51–1.36)	0.47
COPD	n.a.		1.85 (1.14–3.01)	0.01
***Medication at admission*:**				
Beta-blocker	1.44 (0.74–2.82)	0.29	1.28 (0.77–2.13)	0.34
ACEi/ARB	1.36 (0.68–2.70)	0.39	1.52 (0.90–2.55)	0.12
Thiazide diuretic	1.01 (0.36–2.88)	0.98	0.59 (0.25–1.36)	0.21
Loop diuretic	0.82 (0.40–1.67)	0.58	1.92 (1.08–3.43)	0.03
Aldosterone antagonist	1.23 (0.37–4.11)	0.74	1.94 (1.07–3.51)	0.03
***Laboratory findings at admission*:**				
Glucose (mmol/L)	1.02 (0.89–1.18)	0.77	1.04 (0.98–1.10)	0.19
K^+^ (mmol/L)	2.68 (1.24–5.82)	0.01	2.09 (1.39–3.14)	<0.001
Creatinine clearance (mL/min)	0.98 (0.97–1.00)	0.04	0.98 (0.97–0.99)	<0.001
C-reactive protein (mg/L) ^a^	1.14 (0.94–1.38)	0.20	1.26 (1.06–1.50)	0.01
NT-proBNP (pg/mL) ^a^	1.07 (0.85–1.35)	0.58	1.53 (1.24–1.89)	<0.001
hs-TnT (ng/L) ^a^	1.36 (0.89–2.07)	0.16	1.37 (1.10–1.71)	0.01
***Na***^***+***^ ***during hospital stay*:**				
Admission Na^+^ (per mmol/L decrease)*	1.01 (0.95–1.07)	0.77	1.02 (0.96–1.07)	0.60
Admission Na^+^ < 137 mmol/L*	1.00 (0.47–2.15)	1.00	1.85 (1.08–3.16)	0.02
Admission Na^+^ < 130 mmol/L*	1.48 (0.45–4.87)	0.52	0.98 (0.31–3.11)	0.97
Discharge Na^+^ (per mmol/L decrease)	0.97 (0.85–1.12)^†^	0.71	1.00 (0.92–1.109)^†^	0.94
Discharge Na^+^ < 137 mmol/L	1.15 (0.46–2.87)^†^	0.77	1.58 (0.85–2.92)^†^	0.15
ΔNa^+^ (discharge—admission)	1.03 (0.93–1.14) ^†^	0.59	1.03 (0.96–1.10) ^†^	0.48
ΔNa^+^ (discharge—admission) if admission Na^+^ < 137 mmol/L	1.02 (0.89–1.17) ^†^	0.78	0.95 (0.84–1.08) ^†^	0.43
**Significant risk markers after multivariate analysis**				
Age (years)	n.s.		1.04 (1.01–1.08)	0.01
Male	n.s.		0.50 (0.30–0.84)	0.01
Body mass index (Kg/m2)	0.89 (0.83–0.96)	0.002	n.s.	
Mean arterial pressure (per 5 mmHg)	n.s.		0.90 (0.84–0.96)	0.002
History of diabetes mellitus	n.s.		2.99 (1.70–5.28)	<0.001
History of COPD	n.s.		2.61 (1.48–4.59)	0.001
K^+^ (mmol/l)	n.s.		1.98 (1.29–3.03)	0.002
NT-proBNP (pg/ml)^a^	n.s		1.70 (1.35–2.14)	<0.001

Hazard ratios are expressed per unit (yes vs. no for binary variables) unless otherwise specified.

^a^ Log transformed because of severe right-skewed distribution

^†^ Missing = 10–50%

* Na^+^ corrected for hyperglycemia by the Hillier formula [25].

Abbreviations: ACE 2 Study, Akershus Cardiac Examination 2 Study; ACEi, angiotensin-converting-enzyme inhibitor; ARB, angiotensin II receptor blocker; CI, confidence interval; COPD, chronic obstructive pulmonary disease; FEV1, forced expiratory volume in one second; FVC, forced vital capacity; hs-TnT, high sensitivity troponin T; LVEF, left ventricular ejection fraction; n.a., not applicable (missing data > 85% for FEV1 and FVC in acute HF, all subjects with acute exacerbation of COPD had a history of COPD and only 3 of them had never smoked); n.s, not statistically significant; NT-proBNP, N-terminal pro-B-type natriuretic peptide; NYHA, New York Heart Association; vs., versus.

## Discussion

The main findings of the present study are (1) that the prevalence of hyponatremia is similar in patients hospitalized with AECOPD and acute HF, but (2) in contrast to acute HF, hyponatremia is not associated with mortality in AECOPD. These observations suggest that different pathophysiological mechanisms may cause hyponatremia, and that the underlying mechanisms may play a more important role for the outcome than hyponatremia *per se*.

We found that hyponatremia is common in patients hospitalized for acute dyspnea whether the cause of dyspnea is acute HF or AECOPD. This is in accordance with previous studies of the general hospital population [2] and acute HF [3, 4], but sparse data are available concerning the prevalence of hyponatremia in other COPD cohorts. In the literature, we found only one previous study reporting the frequency of hyponatremia in COPD [27]. In that small observational study from 1965, 14 out of 30 patients hospitalized with moderate to severe COPD had serum Na^+^ <136 mmol/L, which results in a prevalence estimate of 47% (95% CI 30–64%), i.e. higher than the prevalence observed in the present study. However, in the previous study heart function was assessed by clinical examination only, which is not very sensitive or specific for heart failure [28], and thus the exclusion of HF subjects may not have been complete. In comparison, the cause of dyspnea in the present study was identified by two independent senior physicians who retrospectively reviewed all medical records from the index hospitalization and follow-up, and this is the recommended method to classify patients with acute dyspnea [29]. In contrast to the previous study, we also corrected Na^+^ for the diluting effect of hyperglycemia that lowers plasma Na^+^ independently of HF and COPD [25]. Ultimately, both studies found hyponatremia to be prevalent in AECOPD, but our results may be more specific for AECOPD-related hyponatremia. Additionally, our study found the prevalence of hyponatremia in AECOPD and acute HF to be in the same range.

In the general population the risk of hyponatremia increases with advancing age and female gender [6]. However, these risk factors are not consistently reported in hospital [2] or HF populations[3, 9, 10]. In the derivation cohort of the present study, only history of hypertension was associated with hyponatremia in COPD patients. This association could be related to anti-hypertensive or diuretic therapy, but we did not find medication to be associated with hyponatremia in our study. Among patients with acute HF, low BMI was independently associated with hyponatremia. Association between hyponatremia and BMI has previously been reported for thiazide-induced hyponatremia [30], but to the best of our knowledge, not in HF-related hyponatremia. Although hyponatremia is considered to reflect the severity of HF [31], we did not find any correlation between hyponatremia and established indices of cardiac function like LVEF, NT-proBNP, and hs-TnT, which suggests that additional factors (e.g. water intake) are important for developing hyponatremia in HF patients.

Hyponatremia is a well-established risk marker in the general population,[6] hospitalized patients [7], and HF patients [3]. In fact, hyponatremia is a predictor of mortality in virtually all previously studied medical conditions [32, 33]. Our study is the first to report the prognostic significance of hyponatremia in AECOPD, but in contrast to previously studied conditions, we did not find any association between hyponatremia and mortality in two independent AECOPD cohorts. Furthermore, we found that the divergent effect of hyponatremia on mortality observed between AECOPD and acute HF subjects is statistically significant. Thus, the prognostic significance of hyponatremia appears to be dependent on the cause of hyponatremia and should be considered in relation to the underlying condition.

The lack of association between hyponatremia and mortality among AECOPD patients in our study could have several explanations. The most likely reason is that factors other than hyponatremia are strongly related to clinical outcomes in AECOPD, including cachexia. In accordance with this theory and previous studies [34, 35], BMI was inversely and independently associated with outcome (Table 2).

The contrasting prognostic impact of hyponatremia in AECOPD and acute HF could also imply disparate mechanism or duration of hyponatremia. Hypoxia, hypercapnia, and acidemia have been suggested as non-osmotic stimuli of vasopressin that may lead to transient hyponatremia during exacerbation of COPD [36–38]. These factors do not necessarily reflect the severity of the underlying chronic disease and could therefore possibly obscure an association between hyponatremia and outcome when patients are included in the acute setting. Arterial blood gasses and pH at admission was not associated with hyponatremia among AECOPD patients in the present study (S1 Table), but as the information regarding oxygen therapy prior to the time of measurement may be incomplete, this question should be explored in additional studies.

Previous studies have found the effect of hyponatremia on mortality to be proportional to the severity of hyponatremia in hospitalized patients [7]. In our study, survival curves stratified by Na^+^ quintiles did not reveal any overall difference or trend for decreasing levels of Na^+^ in AECOPD (S2 Fig). We also explored different cut-offs for hyponatremia that could possibly predict mortality better than Na^+^ < 137 mmol/L, but no Na^+^ cut-off between < 130 mmol/L and < 138 mmol/L was found to be associated with increased mortality by univariate cox regression analysis (S2 Table). However, it should be mentioned that our study is not powered to detect small-scale effects of more severe hyponatremia on mortality as indicated by wide 95% CI for lower Na^+^ cut-offs.

The missing effect of hyponatremia (Na^+^<137 mmol/L) on mortality in AECOPD could potentially also be explained by limited statistical power. Post-hoc power calculations suggest that the combined sample size of the derivation and validation cohort may be insufficient to detect a HR for hyponatremia less than 1.79 with at least 80% statistical power (S3 Table). However, the recommended method to evaluate the limitation of negative results is to interpret the 95% CI for estimates calculated from the actual data [39]. The 95% CI for the effect of hyponatremia indicate that the true HR for hyponatremia in AECOPD can be found between 0.53 and 1.40 in the combined cohort. Accordingly, even though we cannot rule out the possibility that hyponatremia increases the risk of mortality in AECOPD, a HR similar to that found for hyponatremia in acute HF by univariate Cox analysis (1.85) would have been detected. Pertinent to this point; the total lack of divergence between survival curves stratified according to the presence of hyponatremia among the AECOPD patients (Fig 2C) indicates that an effect of hyponatremia on survival in AECOPD is small and most likely of limited clinical relevance.

Our study contrasts with previous studies [3] by not being able to confirm that the association between hyponatremia and mortality in acute HF is independent of conventional risk indices. This could pertain to a lack of statistical power to demonstrate an association in multivariate analysis, or relate to our comprehensive multivariate model that, in contrast to previous reports, include novel cardiac biomarkers (hs-TnT and NT-proBNP) closely associated with outcome in acute HF. However, one previous community-based study of chronic HF found hyponatremia to be associated with mortality after adjustment for NT-proBNP [40], thus the prognostic significance of hyponatremia in chronic HF seems to be strong.

### Strengths and Limitations

The ACE 2 study is a modestly sized prospective study of unselected patients admitted to a single center with acute dyspnea. The single-center design ensured uniform handling of all samples that could improve the accuracy of our results. As no single specific index can be used to diagnose acute HF [29] or AECOPD [21], we stratified subjects by an adjudication committee. This is considered to be the “gold-standard” strategy in order to avoid misclassification, and our adjudication committee classified patients more uniformly than previous adjudication committees in similar studies [41, 42]. The validation cohort based the classification of AECOPD on the diagnosis by the attending physician in charge of the patient, which is less robust compared to an adjudication committee. However, as we find consistent results for hyponatremia across the two populations, we believe misclassification is not a major problem in our study and not likely to influence the results. We also acknowledge that HF and COPD may coexist in a proportion of our patients; i.e. 11% of AECOPD subjects had a history of HF and 46% of acute HF subjects had a history of COPD in the derivation cohort. Thus, assuming that hyponatremia is associated increased mortality in HF, the misclassification of HF or coexistence of HF in AECOPD could lead to a false positive result. However, as hyponatremia was not associated with mortality among AECOPD subjects in our study, a false positive result is not a major concern. Finally, our results are limited to AECOPD by study design, and the association between hyponatremia and mortality in stable-state COPD should be explored in additional studies as information of pre-hospital Na^+^ measurements were not available in our study participants

## Conclusion

Hyponatremia is prevalent in patients hospitalized with AECOPD, but does not predict mortality during follow-up. Additional studies are required to uncover the mechanism of hyponatremia in AECOPD and to explain the diverging prognostic impact of hyponatremia in AECOPD as compared to HF and almost all other previously studied medical disorders.

## Supporting Information

S1 Fig AECOPD validation cohort flow chart. (TIFF)

S2 Fig Kaplan-Meier plots stratified by Na^+^ level. (TIFF)

S1 Table Logistic regression for the presence of hyponatremia in the derivation cohort (ACE 2 study). (PDF)

S2 Table Hyponatremia sensitivity and specificity for predicting death at different Na^+^ cut-offs and corresponding hazard ratios in acute exacerbation of COPD (ACE 2 Study). (PDF)

S3 Table Minimal detectable HR per unit reduction of Na^+^ and for the presence of hyponatremia. (PDF)

S1 Text Subjects and Methods in the ACE 2 study. (PDF)
