# Supplementary figures and images for: Prevalence and Prognostic Significance of Hyponatremia in Patients with Acute Exacerbation of Chronic Obstructive Pulmonary Disease: Data from the Akershus Cardiac Examination (ACE) 2 Study
Source: PLoS One. 2016 Aug 16;11(8):e0161232. doi: 10.1371/journal.pone.0161232 (PMC4987051; doi:10.1371/journal.pone.0161232)

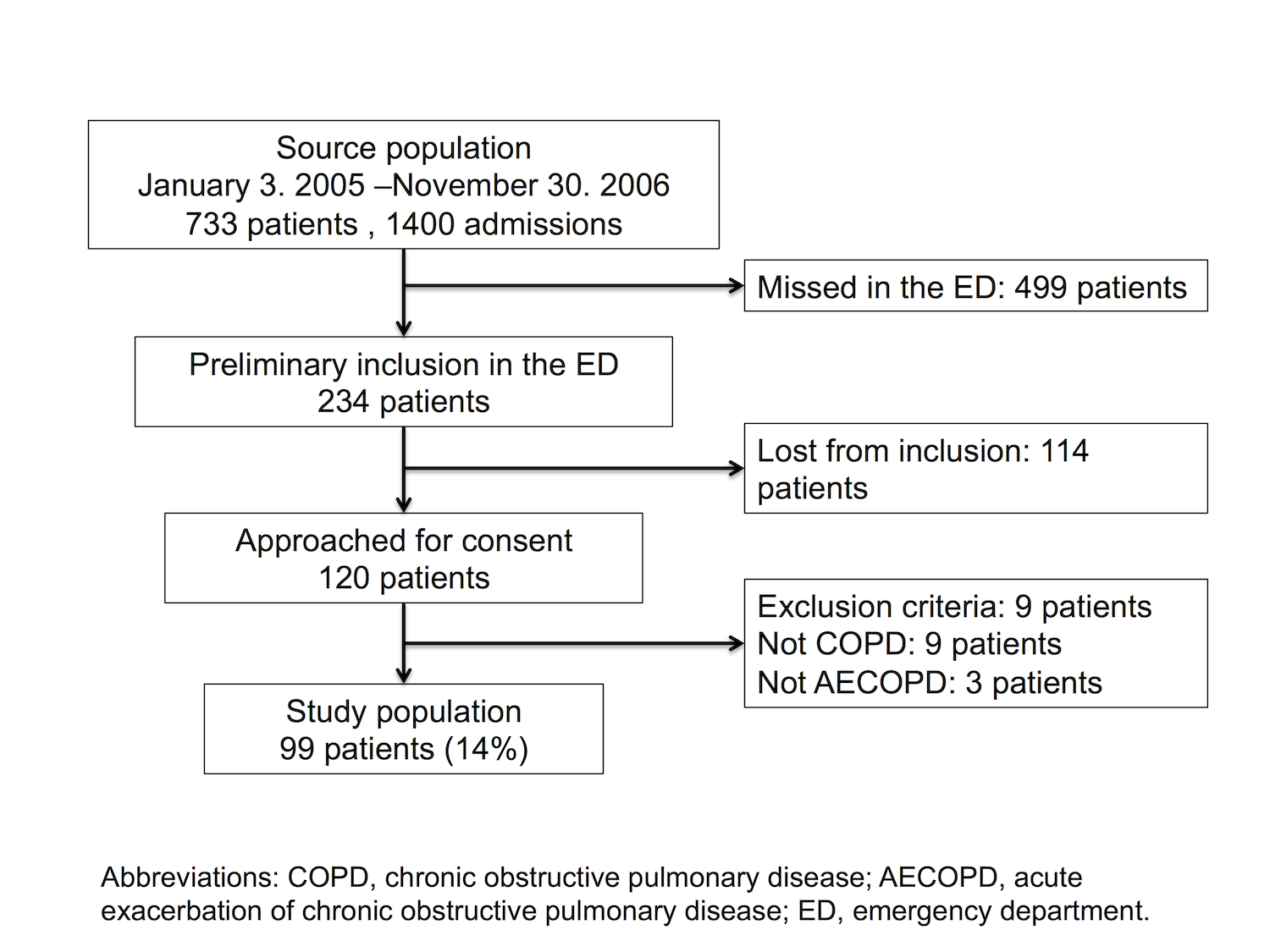

Supplement: S1 Fig — (TIFF) [file pone.0161232.s001.tiff]

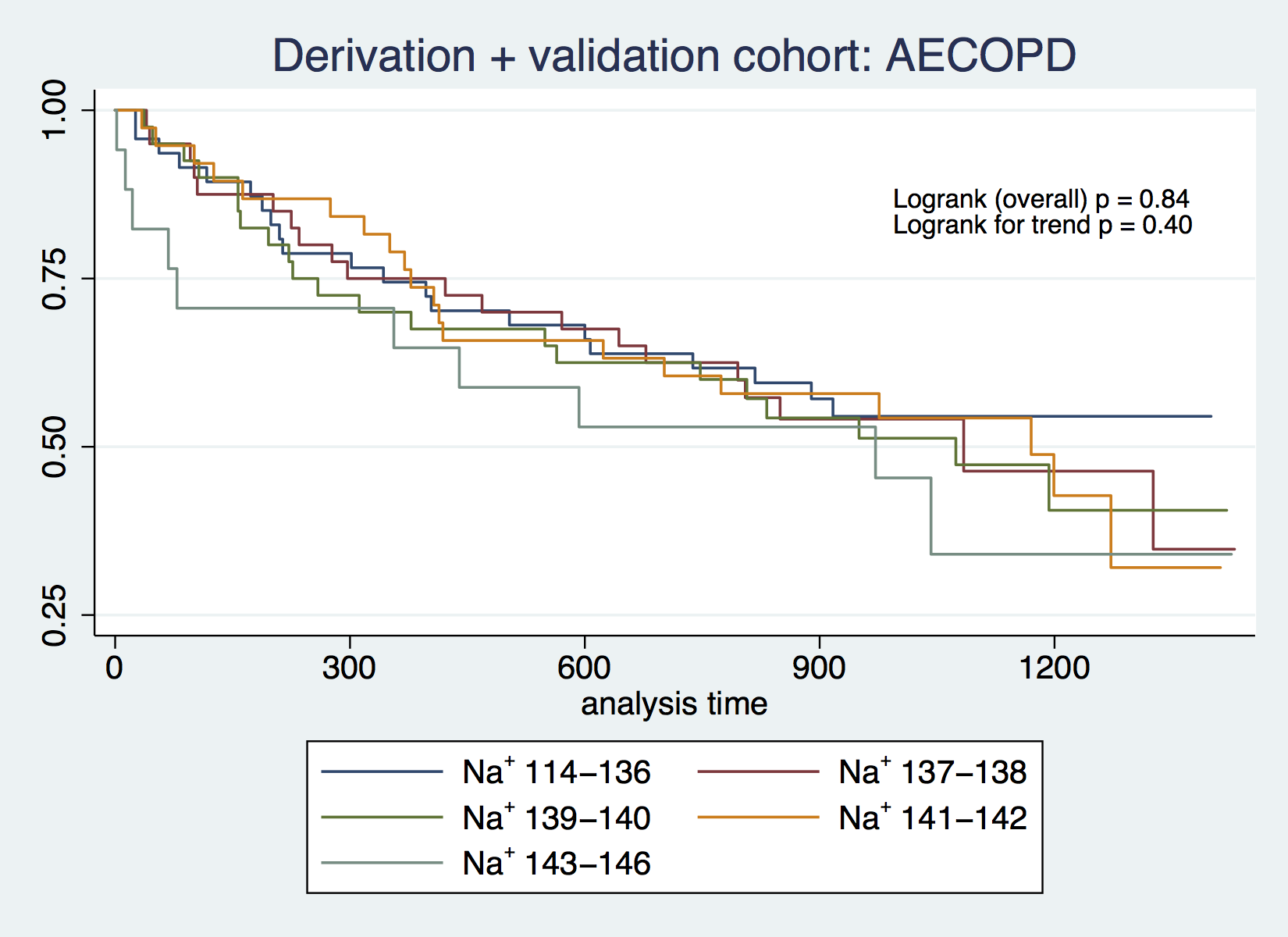

Supplement: S2 Fig — (TIFF) [file pone.0161232.s002.tiff]
