# Supplementary material for: Prevalence and Prognostic Significance of Hyponatremia in Patients with Acute Exacerbation of Chronic Obstructive Pulmonary Disease: Data from the Akershus Cardiac Examination (ACE) 2 Study
Source: PLoS One. 2016 Aug 16;11(8):e0161232. doi: 10.1371/journal.pone.0161232 (PMC4987051; doi:10.1371/journal.pone.0161232)
Supplement: S1 Table — (PDF) [file pone.0161232.s003.pdf]

**S1 Table. Logistic regression for the presence of hyponatremia in the derivation cohort (ACE 2 study)**

| | Patients with acute exacerbation of COPD (n=83) | | Patients with acute heart failure (n=143) | |
|---|---|---|---|---|
| | Odds ratio (95% CI) | *P* | Odds ratio (95% CI) | *P* |
| ***Clinical findings at admission:*** | | | | |
| Age (years) | 1.02 (0.96-1.07) | 0.58 | 1.05 (1.00-1.09) | 0.05 |
| Male sex | 0.72 (0.26-1.97) | 0.52 | 0.27 (0.12-0.63) | 0.003 |
| Body mass index ($Kg/m^2$) | 0.96 (0.88-1.04) | 0.34 | 0.86 (0.78-0.95) | 0.002 |
| Heart rate (per 5 beats/minute) | 1.09 (0.95-1.24) | 0.23 | 0.99 (0.92-1.07) | 0.82 |
| Mean arterial blood pressure (per 5 mmHg) | 1.03 (0.90-1.18) | 0.66 | 0.97 (0.87-1.07) | 0.50 |
| Peripheral edema | 0.54 (0.19-1.57) | 0.26 | 0.64 (0.28-1.44) | 0.28 |
| Pre-hospital $SpO_2$ (%) | 0.99 (0.94-1.05)$^{\dagger}$ | 0.83 | 1.06 (0.85-1.32)$^{\ddagger}$ | 0.61 |
| NYHA class IV vs. II-III | 0.89 (0.33-2.38) | 0.82 | 1.37 (0.61-3.10) | 0.45 |
| ***Heart and lung function:*** | | | | |
| LVEF (%) | 1.05 (0.95-1.15)$^{\dagger}$ | 0.34 | 1.02 (0.99-1.05) | 0.27 |
| $FEV_1$ (per 100 mL) | 0.97 (0.87-1.09) | 0.64 | n.a. | |
| $FEV_1$ % of predicted (per 10 %) | 0.96 (0.71-1.31) | 0.81 | n.a. | |
| $FEV_1$/FVC (per 10%) | 1.04 (0.74-1.46) | 0.81 | n.a. | |
| ***Smoking:*** | | | | |
| Current vs. previous and never | 1.99 (0.73-5.43) | 0.18 | 1.59 (0.62-4.07) | 0.33 |
| Never vs. current and previous | n.a. | | 1.54 (0.64-3.70) | 0.33 |
| ***History of:*** | | | | |
| Diabetes mellitus | 0.32 (0.04-2.68) | 0.29 | 0.69 (0.27-1.76) | 0.44 |
| Heart failure | 0.77 (0.15-4.03) | 0.76 | 0.52 (0.23-1.19) | 0.12 |
| Coronary artery disease | 1.61 (0.57-4.54) | 0.37 | 0.87 (0.38-1.96) | 0.73 |
| Hypertension | 3.07 (1.11-8.50) | 0.03 | 0.84 (0.37-1.91) | 0.68 |
| COPD | n.a. | | 0.78 (0.34-1.81) | 0.57 |
| ***Medication at admission:*** | | | | |
| Beta-blocker | 0.72 (0.26-2.02) | 0.53 | 0.99 (0.43-2.30) | 0.98 |
| ACEi/ARB | 2.16 (0.79-5.91) | 0.14 | 1.91 (0.78-4.67) | 0.16 |
| Thiazide diuretic | 2.04 (0.52-8.04) | 0.31 | 2.22 (0.75-6.53) | 0.15 |
| Loop diuretic | 1.17 (0.42-3.25) | 0.76 | 1.07 (0.44-2.57) | 0.88 |
| Aldosterone antagonist | 1.43 (0.24-8.38) | 0.70 | 2.27 (0.82-6.29) | 0.11 |
| ***Laboratory findings at admission:*** | | | | |
| Arterial pH (per 0.1) | 1.20 (0.45-3.22) | 0.71 | 0.87 (037-2.03) | 0.75 |
| Arterial $pO_2$ (kPa) | 0.88 (0.69-1.12) | 0.29 | 1.23 (1.01-1.51) | 0.04 |
| Arterial $pCO_2$ (kPa) | 1.04 (0.82-1.31) | 0.75 | 0.95 (0.65-1.40) | 0.79 |

| | | | | |
|---|---|---|---|---|
| Glucose (mmol/L) | 0.92 (0.74-1.15) | 0.47 | 0.82 (0.66-1.01) | 0.07 |
| $K^+$ (mmol/L) | 1.34 (0.50-3.60) | 0.57 | 1.43(0.71-2.86) | 0.32 |
| Creatinine clearance (mL/min) | 1.00 (0.98-1.02) | 1.00 | 0.99 (0.97-1.00) | 0.045 |
| C-reactive protein (mg/L)[a] | 1.29 (0.95-1.75) | 0.11 | 1.05 (0.80-1.38) | 0.75 |
| NT-proBNP (pg/mL) [a] | 1.24 (0.86-1.78) | 0.25 | 1.09 (0.80-1.48) | 0.59 |
| hs-TnT (ng/L) [a] | 0.82 (0.45-1.48) | 0.51 | 1.25 (0.83-1.88) | 0.28 |
| **Significant risk factors after multivariate analysis** | | | | |
| History of hypertension | 3.07 (1.11-8.50) | 0.03 | n.s. | |
| Body mass index (Kg/m2) | n.s. | | 0.83 (0.75-0.93) | 0.001 |

Odds ratios are expressed per unit (yes vs. no for binary variables) unless otherwise specified.

[a] Log transformed because of severe right skew; † Missing = 10-50%; ‡ Missing = 51-85%. Hyponatremia is defined as $Na^+ < 137$ mmol/L after correction for hyperglycemia by the Hillier formula.

Abbreviations: ACE 2 Study, Akershus Cardiac Examination 2 Study; ACEi, angiotensin-converting-enzyme inhibitor; ARB, angiotensin II receptor blocker; CI, confidence interval; COPD, chronic obstructive pulmonary disease; FEV1, forced expiratory volume in one second; FVC, forced vital capacity; hs-TnT, high sensitivity troponin T; LVEF, left ventricular ejection fraction; n.a., not applicable (missing data > 85% for FEV1 and FVC in acute HF, all subjects with acute exacerbation of COPD had a history of COPD and only 3 of them had never smoked ); n.s., not statistically significant.; NT-proBNP, N-terminal pro-B-type natriuretic peptide; NYHA, New York Heart Association; $pCO_2$, partial pressure of carbon dioxide; $pO_2$, partial pressure of oxygen; $SpO_2$, peripheral capillary oxygen saturation; vs., versus.
