# Supplementary material for: Prevalence and Prognostic Significance of Hyponatremia in Patients with Acute Exacerbation of Chronic Obstructive Pulmonary Disease: Data from the Akershus Cardiac Examination (ACE) 2 Study
Source: PLoS One. 2016 Aug 16;11(8):e0161232. doi: 10.1371/journal.pone.0161232 (PMC4987051; doi:10.1371/journal.pone.0161232)
Supplement: S2 Table — (PDF) [file pone.0161232.s004.pdf]

S2 Table. Hyponatremia sensitivity and specificity for predicting death at different Na^+ cut-offs and corresponding hazard ratios in acute exacerbation of COPD (ACE 2 Study)

**Na^+ cut-off**	***Sensitivity (%)***	***Specificity (%)***	***Correctly classified (%)***	**Youden's index**	**Hazard ratio † (95% CI)**	***P***
Na^+ < 138 mmol/L	40.0	60.4	51.8	0.04	1.02 (0.52-2.03)	0.95
Na^+ < 137 mmol/L	25.7	72.9	53.0	-0.01	1.00 (0.47-2.15)	1.00
Na^+ < 136 mmol/L	25.7	77.1	55.4	0.03	1.16 (0.54-2.48)	0.71
Na^+ < 135 mmol/L	25.7	83.3	59.2	0.09	1.52 (0.71-3.26)	0.62
Na^+ < 134 mmol/L	22.9	87.5	60.2	0.10	1.58 (0.71-3.50)	0.26
Na^+ < 133 mmol/L	14.3	91.7	59.0	0.06	1.29 (0.50-3.34)	0.56
Na^+ < 132 mmol/L	8.6	93.8	57.8	0.02	1.11 (0.34-3.66)	0.86
Na^+ < 131 mmol/L*	8.6	93.8	57.8	0.02	1.11 (0.34-3.66)	0.86
Na^+ < 130 mmol/L	8.6	95.8	59.0	0.04	1.48 (0.45-4.87)	0.52

* None of the subjects had Na^+ = 131 mmol/L, thus the results are the same as for 132 mmol/L; † Hazard ratio by Cox regression analysis
