# Supplementary material for: Prevalence and Prognostic Significance of Hyponatremia in Patients with Acute Exacerbation of Chronic Obstructive Pulmonary Disease: Data from the Akershus Cardiac Examination (ACE) 2 Study
Source: PLoS One. 2016 Aug 16;11(8):e0161232. doi: 10.1371/journal.pone.0161232 (PMC4987051; doi:10.1371/journal.pone.0161232)
Supplement: S3 Table — (PDF) [file pone.0161232.s005.pdf]

S3 Table. Minimal detectable HR per unit reduction of Na^+ and for the presence of hyponatremia

Variable	Power (%)	Hazard ratio*			
		AECOPD Derivation N= 83	AECOPD Validation N=99	AECOPD Derivation + validation N=182	Acute HF N=143
Na^+ (per mmol/L decrease)	50	1.070	1.059	1.044	1.061
	60	1.080	1.067	1.050	1.069
	70	1.090	1.076	1.056	1.078
	80	1.102	1.086	1.064	1.088
	90	1.119	1.100	1.074	1.102
$Na^+ < 137$ mmol/L	50	1.942	1.678	1.502	1.621
	60	2.116	1.794	1.583	1.726
	70	2.320	1.927	1.675	1.845
	80	2.583	2.095	1.789	1.995
	90	2.998	2.353	1.960	2.224

*Calculated Hazard ratios are based on actual sample size, mortality rate, and standard deviation for each cohort, and a two-sided significance level of α=0.05
