# Supplementary material for: Prevalence and Prognostic Significance of Hyponatremia in Patients with Acute Exacerbation of Chronic Obstructive Pulmonary Disease: Data from the Akershus Cardiac Examination (ACE) 2 Study
Source: PLoS One. 2016 Aug 16;11(8):e0161232. doi: 10.1371/journal.pone.0161232 (PMC4987051; doi:10.1371/journal.pone.0161232)
Supplement: S1 Text — (PDF) [file pone.0161232.s006.pdf]

Subjects and Methods in the ACE 2 study

Supplemental material
to

Prevalence and prognostic significance of hyponatremia in patients with acute exacerbation of chronic obstructive pulmonary disease: data from the Akershus Cardiac Examination (ACE) 2 Study

Jacob A. Winther[1,2*], Jon Brynildsen[1,2], Arne Didrik Høiseth[1,2], Ivar Følling[1,2], Pål H. Brekke[1,2,#a], Geir Christensen[2,3], Tor-Arne Hagve[2,4], Joseph G. Verbalis[5], Torbjørn Omland[1,2], Helge Røsjø[1,2]

[1] Division of Medicine, Akershus University Hospital, Lørenskog, Norway

[2] Institute of Clinical Medicine, University of Oslo, Oslo, Norway

[3] Institute for Experimental Medical Research, Oslo University Hospital, Ullevål, Oslo, Norway

[4] Division of Diagnostics and Technology, Akershus University Hospital, Lørenskog, Norway

[5] Division of Endocrinology and Metabolism, Georgetown University Medical Center, Washington, DC, USA

[#a] Current address: Division of Medicine, Oslo University Hospital, Oslo, Norway

Akershus Cardiac Examination (ACE) 2 Study

In total, 314 consecutive patients with the primary complaint of dyspnea admitted to the Division of Medicine at Akershus University Hospital, a Norwegian teaching hospital with a catchment area of 460 000 people, were included in the Akershus Cardiac Examination (ACE) 2 Study [1]. Dedicated study personnel, who attended all briefings in between sets, identified eligible patients in cooperation with medical interns and residents working in the Emergency Department (ED). Patient inclusion occurred between 8 am and 2 pm Monday to Thursday from June 2009 until November 2010. Patients with the shortest time from admission were approached first. The eligibility criteria were dyspnea considered as the primary cause for hospitalization by the ED physician, age ≥18 y, and less than 24 hours from hospital admission to study inclusion. Exclusion criteria were dementia or other causes precluding informed patient consent, disseminated malignant disease, acute myocardial infarction or coronary intervention, and major surgery within the last 2 weeks. As this study was designed to assess the diagnostic and prognostic value of biomarkers, patients with hemoglobin levels <10 g/dL or incomplete study baseline blood sampling due to technical issues were excluded.

Data collection

Clinical information was obtained directly from the ED physicians at the briefings in between sets by the use of a standardized questionnaire. The questionnaire included questions on the duration of dyspnea prior to admission, clinical findings including respiratory frequency, peripheral edema, cyanosis, raised jugular venous pressure, and ECG pathology. Furthermore, the ED physicians were requested to classify the patients according to the New York Heart Association (NYHA) functional class. Pre-hospital capillary oxygen saturation (SpO_2) was collected from hospital or ambulance records where available. Blood pressure, heart rate, and body temperature on hospital admission were collected from patient records. Finally, using a more comprehensive questionnaire, clinical information was retrieved directly from patients by dedicated study personnel. This second questionnaire included questions pertaining to clinical status prior to admission, medical history, co-morbidities, and smoking status. Previous medical history and medication were checked against medical records. Height and weight were collected directly from the patient or from the patient records. Body mass index (BMI) was calculated as weight (Kg)/[height (m)]^2. Mean arterial blood pressure (BP) was calculated by the formula: [(2 x diastolic BP)+systolic BP] / 3. LVEF was recorded from echocardiography reports, and forced expiratory volume of the first second (FEV1) and forced vital capacity (FVC) from spirometry results if available.

Adjudication of diagnosis

The final diagnoses of the index hospitalizations was determined by two independent senior physicians, who reviewed all medical records, including follow-up data with a median time from hospitalization to adjudication of diagnosis of 464 (quartile [Q] 1-3 304-705) days. The adjudication committee had no knowledge of cardiac biomarker levels as measured within the context of the ACE 2 Study, but had access to all other information, including cardiac biomarker measurements that were ordered by the treating physicians. The acute heart failure diagnosis was based on the European Society of Cardiology criteria, requiring typical signs and symptoms of HF and objective evidence of structural or functional myocardial abnormality [2]. The diagnosis of acute exacerbation of COPD was based on the criteria defined by the Global initiative for Chronic Obstructive Lung Disease (GOLD) group [3] : $FEV_1/FVC<70\%$ and no significant improvement by inhalation therapy during pulmonary testing, acute worsening of the patient's symptoms (dyspnea, cough and/or sputum production) that is beyond day-to-day variation and that leads to a change in medication. In the event that a subject fulfilled the criteria of both acute HF and AECOPD, the the most probable cause of the index hospitalization was diagnosed.

Biochemical analysis

Study blood sampling was performed by venipuncture; the blood was immediately put on ice, and processed in a uniform matter throughout the study period. NT-proBNP levels were measured in EDTA plasma samples obtained <24 h after hospital admission by personnel with no knowledge of patient diagnosis (proBNP II assay, Roche Diagnostics, Penzberg, Germany). Troponin T (TnT) levels were measured by a high-sensitivity (hs) assay (Troponin T hs STAT assay, Roche Diagnostics) in serum samples obtained in parallel to EDTA samples for NT-proBNP measurements. In plasma, the Na^+ level is inversely correlated to the glucose levels due to the osmotic properties of glucose [4]. Thus, we decided to correct Na^+ for the diluting effect of glucose before the diagnosis of hyponatremia was established. Several correction factors have been suggested in the literature, but the Hillier formula appears to provide the best overall correction [5]. The Hillier formula increases Na^+ by 2.4 mmol/L for every 5.6 mmol/L (100 mg/dL) increase in glucose above 5.6 mmol/L. Arterial blood gas (ABG) measurements: pH, pCO_2, and pO_2, were retrospectively retrieved from hospital records. Arterial blood was drawn by the ED physician and analyzed in the ED department as part of the standard work-up at admission. If several ABG measurements from admission were available, the first measurement was included. If the ED physician indicated that the measurements were performed on venous blood the result was not included.

Statistical analysis

Continuous variables with normal distribution are reported as mean (± standard deviation [SD]), while variables with non-normal distribution (as assessed by histograms and the Kolmogorov-Smirnov test) are presented as median (quartile [Q] 1-3). As the relationship between plasma Na^+ and mortality appear to be U-shaped[6] and may not increase proportionally with severity of hyponatremia,[7] we assessed the effect of Na^+ on outcome by stratifying patients into hyponatremia or no hyponatremia (i.e. a binary variable). We explored risk markers of hyponatremia by binary logistic regression models and present odds ratios (OR) with 95% confidence intervals (CI). Statistically significant variables by univariate logistic regression and Cox regression analysis were entered into the respective multivariate model by stepwise forward selection based on the likelihood ratio (LR) criterion. Age and gender have previously been found to be associated with hyponatremia[6] and were therefore included in the group of variables eligible for forward selection irrespective of the level of significance by univariate analysis. The distribution of NT-proBNP, hs-TnT, and CRP were severely right-skewed and thus transformed by the natural logarithm. The resulting HR and OR of these variables in regression analysis is thus reported as per 1 unit increase in log-transformed biomarker level. NYHA functional class was dichotomized and included in the analysis as class IV vs. class II-III. ABG measures were retrieved retrospectively and missing in a substantial proportion of study participants and therefore only included in the logistic regression analysis. Goodness of fit for continuous variables in logistic regression models was assessed by the Hosmer-Lemeshow test. The validity of proportional hazards assumption in Cox regression was tested on the basis of Schoenfeld residuals.

References

1. Rosjo H, Dahl MB, Jorgensen M, Roysland R, Brynildsen J, et al. (2015) Influence of Glycosylation on Diagnostic and Prognostic Accuracy of N-Terminal Pro-B-Type Natriuretic Peptide in Acute Dyspnea: Data from the Akershus Cardiac Examination 2 Study. Clin Chem 61: 1087-1097.

2. McMurray JJ, Adamopoulos S, Anker SD, Auricchio A, Bohm M, et al. (2012) ESC Guidelines for the diagnosis and treatment of acute and chronic heart failure 2012: The Task Force for the Diagnosis and Treatment of Acute and Chronic Heart Failure 2012 of the European Society of Cardiology. Developed in collaboration with the Heart Failure Association (HFA) of the ESC. Eur Heart J 33: 1787-1847.

3. (2011) Global Initiative for Chronic Obstructive Lung Disease (GOLD). Global strategy for Diagnosis, Management and Prevention of Chronic Pulmonary Disease.

4. Seldin DW, Tarail R (1949) Effect of hypertonic solutions on metabolism and excretion of electrolytes. Am J Physiol 159: 160-174.

5. Hillier TA, Abbott RD, Barrett EJ (1999) Hyponatremia: evaluating the correction factor for hyperglycemia. Am J Med 106: 399-403.

6. Mohan S, Gu S, Parikh A, Radhakrishnan J (2013) Prevalence of hyponatremia and association with mortality: results from NHANES. Am J Med 126: 1127-1137 e1121.

7. Chawla A, Sterns RH, Nigwekar SU, Cappuccio JD (2011) Mortality and serum sodium: do patients die from or with hyponatremia? Clin J Am Soc Nephrol 6: 960-965.
